# Current diagnostic tools and management modalities of *Nocardia* keratitis

**DOI:** 10.1186/s12348-020-00228-w

**Published:** 2020-12-02

**Authors:** Mohammad Soleimani, Ahmad Masoumi, Sadegh Khodavaisy, Mostafa Heidari, Ali A. Haydar, Alireza Izadi

**Affiliations:** 1 Ocular Trauma and Emergency Department, Farabi Eye Hospital, Tehran University of Medical Sciences, Tehran, Iran; 2 Department of Medical Parasitology and Mycology, School of Public Health, Tehran University of Medical Sciences, Tehran, Iran

**Keywords:** Nocardia, Keratitis, Corneal ulcer, Confocal microscopy, Amikacin, Keratoplasty, Actinomycetes, Ocular trauma

## Abstract

*Nocardia* species are an uncommon but important cause of keratitis. The purpose of this review is to discus previous published papers relation to the epidemiology, etiology, diagnosis and management of Nocardia keratitis. *Nocardia asteroides* is the most frequently reported from Nocardia keratitis. Pain, photophobia, blepharospasm and lid swelling are mainly clinical manifestations. Usual risk factors for Nocardia keratitis are trauma, surgery, corticosteroids, and contact lens wear. Several antibiotics were used for treatment of Nocardia infection but according to studies, topical amikacin is the drug of choice for Nocardia keratitis. Topical steroid should not prescribe in these patients. In conclusion, although *Nocardia* keratitis is rare, early diagnosis and treatment are essential to prevent any scar formation and preserve a good visual acuity.

## Background

*Nocardia* species are Gram-variable, obligate aerobic, non-motile, branching, beaded filamentous, weakly acid-fast bacilli, of the family *Actinomycetaceae*. *Nocardia* are ubiquitous soil saprophytes that are transmitted by either airborne or direct cutaneous inoculation route [1].

*Nocardia* are a rare cause of infectious keratitis. The diagnosis of *Nocardia* keratitis is challenging because they are not commonly encountered in clinical practice and mainly mimic fungal keratitis [2]. Typically, patients present after being treated with multiple empiric antibiotics for bacterial and/or fungal keratitis which may not have an appropriate effect on *Nocardia* species. Thus, delayed diagnosis and exacerbated ocular complications are common. Topical corticosteroids were associated with worse visual outcomes [3]. *Nocardia* keratitis clinical presentation includes ocular pain—out of proportion to exam—photophobia, blepharospasm, and eyelid edema. The slit-lamp examination reveals a classic wreath-like stromal infiltrates with satellite lesions [4, 5]. Historically, sulfonamides like sulfacetamide and sulfamethoxazole-trimethoprim were used to treat *Nocardia* keratitis. Currently, the first-line treatment of *Nocardia* keratitis is topical amikacin [6].

## Main text

A review of the literature conducted based on the database sources such as MEDLINE, web of scence, Scopus, PubMed and Google scholar from 2000 up to now. We searched all valuable and relevant information considering the epidemiology, etiology, diagnosis and management of Nocardia keratitis. The following keywords were used: *Nocardia*, keratitis, corneal ulcer, confocal microscopy, amikacin, keratoplasty, actinomycetes, and ocular trauma.

## Bacteriology

The genus *Nocardia* is an aerobic Actinomycete known to cause disseminated and focal infections in humans. *Nocardia* was first described in 1888 by Edmond Nocard, a veterinarian, who isolated the organism from a cattle with bovine farcy [1]. In 1889, Trevisan named this strain as *Nocardia farcinica* [6]. *Nocardia asteroides* was isolated from a human brain abscess by Eppinger, in 1890, who named it *Claothrix asteroides* and subsequently was renamed as *Nocardia asteroides* by Blanchard in 1896 [7]. Recently, Conville et al. reviewed the taxonomy of *Nocardia*. The authors counted 92 recognized species of the *Nocardia* genus of which 54 species are clinically significant [7]. The following species were isolated from ocular tissues: *N. abscessus, N. amikacinitolerans, N. amamiensis, N. beijingensis, N. brasiliensis, N. cyriacigeorgica, N. farcinica, N. exalbida, N. kruczakiae, N. otitidiscaviarum, N. puris, N. shinanonensis, N. transvalensis,* and *N. thailandica* [7]. *Nocardia asteroides* is the most frequently *Nocardia* taxon isolated from human specimens [8].

## Epidemiology

*Nocardia spp.* has been considered as a relatively rare cause of bacterial keratitis in recent years [6]. Current knowledge about the incidence of Nocardia keratitis is based on sporadic case reports and case series from various countries [3, 9]. Previous studies from Nepal [10], India (Heydarabad) [11] and India (Tamil Nadu) [12] reported that Nocardia constituted 0.3%, 1.7% and 4.2% of all bacterial isolates from cases of keratitis respectively.

## Risk factors

Trauma is the most common predisposing factor for *Nocardia* keratitis [5]. The other usual risk factors are surgery, corticosteroids, and contact lens wear [5]. Javadi et al. reported an outbreak of *Nocardia* keratitis post photorefractive keratectomy (PRK) in 4 eyes operated by one surgeon, the same day [13]. Inadequate sterilization was concluded as the most probable cause of the outbreak. Many studies have described *Nocardia* keratitis post PRK and laser in situ keratomileusis (LASIK) [14, 15]. Troumani et al. documented a case of *N. abscessus* keratitis following a vegetal trauma [16]. Multiple case reports have identified the chronic wearing of contact lens as a predisposing factor for *Nocardia* keratitis (*N. arthritidis* [17], *N. amikacinitolerans* [1], *N. farcinica* [18], and *N. exalbida* [19]). Bharathi et al. studied 31 patients with *Nocardia* keratitis in south India and found that corneal injuries with soil and sand, agricultural works, corneal loose sutures, previous ocular surgery, and living in rural areas are the most important risk factors for *Nocardia* keratitis [20]. The authors found a male predominance. Another study reported a corneal ulcer with *N. nova* 48 days after uncomplicated cataract surgery [21]. The authors treated the ulcer successfully with oral co-trimoxazole and topical amikacin and moxifloxacin. Corticosteroid usage is another predisposing risk factor for *Nocardia* keratitis. A two-week treatment course of topical prednisolone acetate 1%, after endothelial corneal graft rejection, was complicated by *N. asteroides* keratitis [22]. This corticosteroid induced ulcer was successfully treated with topical amikacin and ofloxacin. Other predisposing factors for *Nocardia* keratitis include diabetes (*N. brasiliensis*) [23], penetrating corneal injury [24] and travelling to Asia (*N. transvalensis*) [25].

## Clinical examination

### Clinical manifestations

**Fig. 1:**
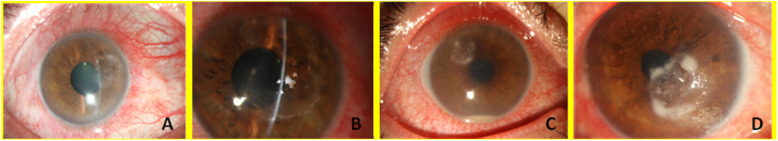
*Nocardia* keratitis (**a**, **b**, **c** and **d)**. Typical superficial patchy, white and pin-head infiltrates in a wreath-like pattern. Active lesion margins are seen

*Nocardia* keratitis presents with pain, photophobia, blepharospasm, and lid swelling [6]. The clinical course is usually slow, and the patient has good visual acuity at presentation [26]. Fine papillary reaction may exist on the conjunctiva**.** The slit-lamp examination shows a patchy, white, pin-head infiltrates in the anterior corneal stroma arranged in a wreath-like pattern (Fig. 1) [17]. A mid-peripheral and paracentral or central corneal epithelial defect (CED) associated with stromal edema can be seen [27]. Stromal melting with fluffy and feathery margins can mimics fungal keratitis [15]. Additional associated findings include moderate anterior chamber reaction, hypopyon, satellite lesions, Descemet folds, and diffuse keratic precipitates (KPs) [22]. Mittal et al. reported a case of *Nocardia* sclerokeratitis presented with anterior stromal plaque-like infiltrations, conjunctival injection, and limbal thickening [28]. The feathery margins of the ulcer mimicked fungal keratitis. Smear and culture revealed *Nocardia* species [28]. Jain et al. described a case of *Nocardia* keratitis induced by topical prednisolone acetate 1% [22]. The following signs appeared after 2 weeks of corticosteroids treatment: conjunctival congestion, graft edema with Descemet folds, white patchy granular superficial infiltrates in the supranasal cornea, and a 1 mm hypopyon. Smear and culture exhibited thin, Gram positive, beaded branching filaments characteristic of *Nocardia asteroids* [22]*.*

### Confocal microscopy

**Fig. 2:**
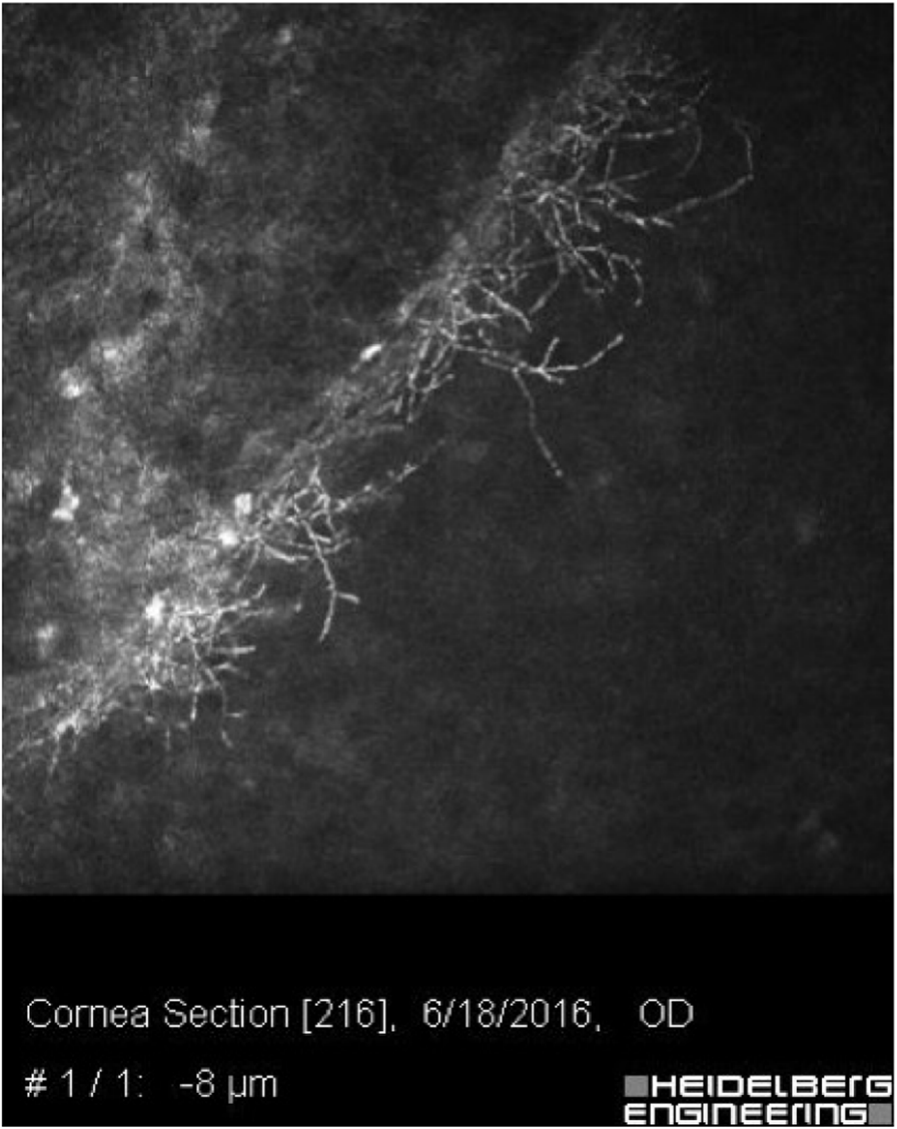
A typical confocal scanning of an eye with *Nocardia* keratitis. Fine branching and interlocking elements are seen

Confocal microscopy is a non-invasive, in vivo modality useful in evaluating normal and pathologic cornea. This device benefits from a lateral resolution of 1 μm and a depth of field of 10 μm. In addition to *Nocardia* keratitis, acanthamoeba and fungal keratitis can also be detected by confocal microscopy. Bacteria are not detected by confocal microscopy because of their small size, however, *Nocardia* can be seen due to their filamentous structure. *Nocardia* will appear as multiple, thin (< 1 μm), short, beaded filamentous structures with right angled branching (Fig. 2) [29]. The round to oval bright structures surrounding the organism represent inflammatory cells. The hyperreflective filaments of *Nocardia* are thinner than fungal hyphae and are best visualized at the margins of the infiltrates [14, 30].

## Diagnostic methods

### Laboratory diagnosis

The diagnosis of *Nocardia* keratitis is often delayed and complicated by long-term inappropriate treatments which lead to corneal scarring and poor visual acuity. Precise history taking and succinct clinical examination can prompt the diagnosis before any laboratory diagnostic evaluation. Good communication between physician and microbiologist is crucial for obtaining and transferring the samples properly and making an accurate diagnosis. The corneal ulcer should be scraped by a spatula or blade for smear and culture preparations [5]. Avoid sampling the conjunctiva and eyelid margins—which exhibit normal flora—is crucial for correct diagnosis [31]. Repeated scrapping should be collected for at least three smears and inoculation of different culture media—growing aerobic and anaerobic bacteria, fungi, and acanthamoeba [6].

### Microscopic evaluation

**Fig. 3:**
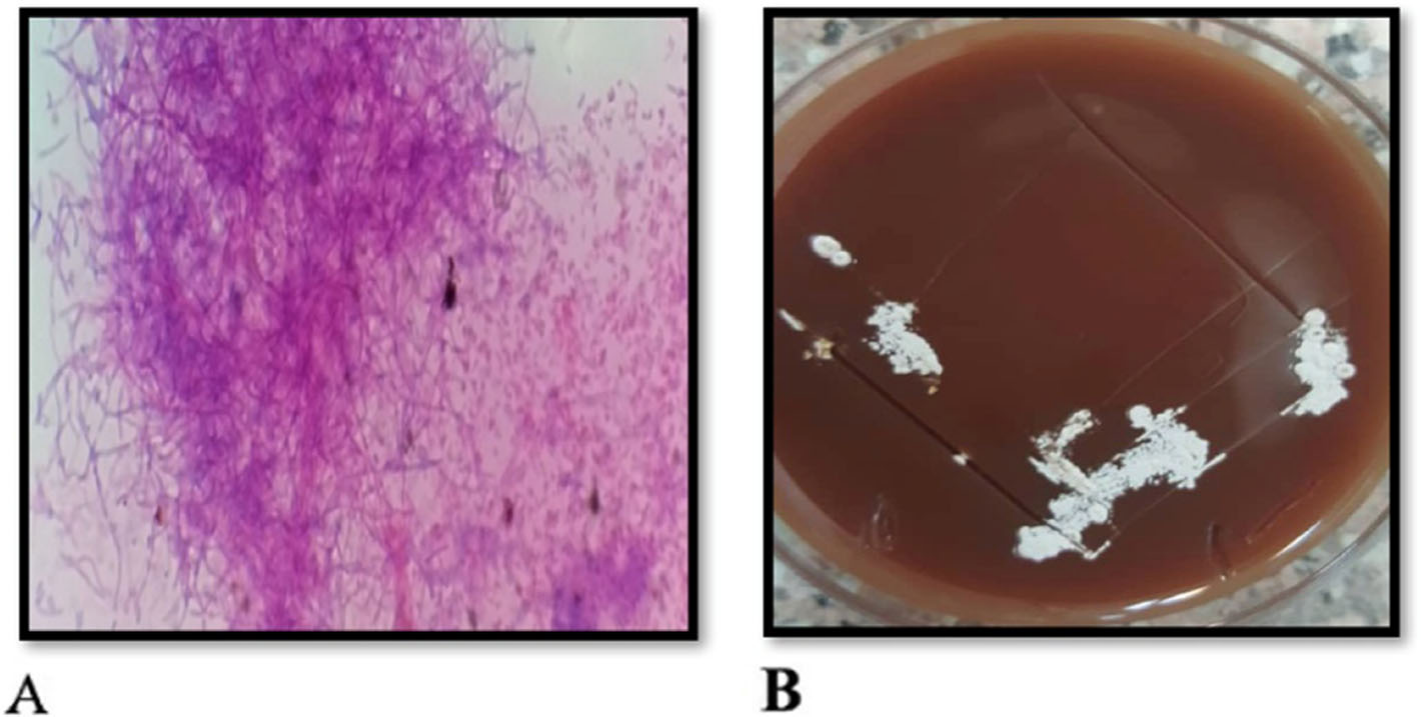
**a** The modified Ziehl-Neelsen or Kinyoun stain method. *Nocardia* isolated from a corneal ulcer showing acid-fast filaments (magnification × 400). **b** Blood agar inoculated with corneal scrapings showing white, dry and chalky colonies

The collected corneal specimens are smeared, stained, and then examined under a light microscope. Staining methods include Gram stain, Giemsa stain, and 10% potassium hydroxide with calcofluor white—superior for detecting *Nocardia* species [20]. Nocardia are beaded Gram-positive, filamentous organisms, weakly acid fast that are best stained with the modified Ziehl-Neelsen or Kinyoun stain method [32] (Fig. 3a). Branching at right angle is a suggestive feature of *Nocardia asteroids* [33]. Actinomycetes, in contrast to fungi, do not fluoresce under ultraviolet (UV) illumination in a calcofluor white staining. All actinomycetes stain black in the Gomori methenamin staining (GMS). The weakly acid-fast characteristic of *Nocardia* species distinguishes them from other actinomycetes. Unlike mycobacteria, *Nocardia* do not resist decolorization with 20% sulfuric acid on Zeihl-Neelsen stain [34]. Histopathological examination reveals scattered stromal necrosis with acute and chronic inflammation [13].

### Culture

*Nocardia* grow slowly in culture but they are not fastidious. *Nocardia* species can be cultured on most available non-selective media. The selective media Thayer-Martin agar with antibiotics can increase the yield especially when specimens are contaminated with normal flora [1]. Typically, growth takes 3–5 days on nonselective agars, although longer inoculation period may be required to achieve a size of 5–10 mm [6]. The filamentous colonies are smooth and moist, and have a chalky, mat or velvety appearance [17] (Fig. 3a and b). The colonies can also exhibit a yellowish color [6]. The filaments will progressively break into coccobacillary elements. Branching may or may not be seen. *Nocardia* are not a part of the ocular or respiratory normal flora—their isolation is considered significant.

### Biochemical methods

*Nocardia* species can be differentiated by various biochemical tests: hydrolysis of amino acids such as adenine, casein, tyrosine, xanthine, and hypoxanthine, decomposition of urea, utilization of citrate, production of nitrate reductase, growth at 45 °C, and producing acid from carbohydrates such as glucose, maltose, lactose, galactose, salicin, xylose, raffinose, arabinose, rhamnose, sorbitol, and sucrose [35]. Conventional methods appear to be unreliable and limited in identifying different *Nocardia* species. Biochemical tests are time consuming and expertise demanding. Only few *Nocardia* species, such as *N. brasiliensis*, *N. farcinica*, and *N. pseudobrasiliensis* can be accurately identified by biochemical tests [35].

### Molecular diagnosis

Molecular diagnostic methods, including gene sequencing, have identified numerous new species of *Nocardia*. Brown et al. introduced a 314-bp DNA fragment tailored to identify *N. farcinia* by PCR assay [36]. Patel et al. investigated the ability of 500-bp 16S rRNA gene sequencing to identify most species of aerobic actinomycetes [37]. The sensitivity and specificity of this method were reported to be 88% and 76%, respectively [38]. Though, the ATCC 19247 strain sequence of *N. asteroides* does not identify any taxa with clinical importance. The PCR-based hsp65 gene sequencing can isolate the species from ocular Nocardiosis [39]. This sequencing can be helpful in identifying *N. arthritidis*—commonly involved in *Nocardia* keratitis, and *N. neocaledoniens*—a causative agent of conjunctivitis. Recently, the use of next-generation sequencing (NGS) has been suggested for timely diagnosis of nocardiosis [40]. Nucleic acid amplification methods are expensive and rarely available. Their usage is limited for atypical and challenging cases or for investigation purposes.

## Treatment

Traditionally, Sulfonamides were the treatment of choice for *Nocardia* keratitis. Trimethoprim-sulfamethoxazole showed superiority to trimethoprim alone or sulfacetamide in clearance of *Nocardia* [41]. Several antibiotics were implemented in the treatment of ocular Nocardiosis including topical chloramphenicol [27], gentamicin [42], polymyxin B sulfate/trimethoprim, amikacin, ciprofloxacin, levofloxacin, moxifloxacin, ofloxacin, gatifloxacin [28], tobramycin, and imipenem [15], and oral clarithromycin [17]. Resistance to tobramycin [43], ciprofloxacin [43], trimethoprim [44], vancomycin [19], clarithromycin [45], and amikacin [1, 46] have been reported. Currently, amikacin is the treatment of choice for ocular *Nocardiosis* [45, 46]. Trimethoprim-sulfamethoxazole can be added to the therapy. Adjuvant use of corticosteroid in bacterial keratitis including *Nocardia* keratitis is still controversial. The Steroids for Corneal Ulcers Trial (SCUT), a randomized multicenter clinical trial, compared prednisolone sodium phosphate 1% to placebo as adjuvant therapies for bacterial corneal ulcers treatment. After 3-month, the authors didn’t find any significant difference in the best corrected visual acuity (BCVA) of both groups [47]. The eyes with *Nocardia* keratitis treated with corticosteroids showed an average of 0.4 mm increase in the infiltrate or scar size after 3-month (*P* = 0.03) [3, 47]. *Nocardia* keratitis has a good prognosis if treated promptly. Lalitha et al. demonstrated that the best visual outcome in *Nocardia* keratitis will be achieved if treatment begins within 15 days of ulcer onset [9]. Delays in diagnosis and treatment of *Nocardia* keratitis result in irreversible scarring and necessitate a surgical management. Therapeutic lamellar keratectomy, penetrating keratoplasty, and conjunctival flap are some of the surgical options [6]. Rahimi et al. used an adjuvant amniotic membrane transplantation (AMT) for persistent epithelial defect (PED) and deep corneal vascularization in *Nocardia* keratitis extending to limbus [48]. PED resolved, yet corneal vascularization persisted despite AMT therapy. Recently, Shah et al. reported a case of multi-drug resistant *Nocardia* keratitis resistant to both amikacin and co-trimoxazole [49]. The authors treated the ulcer with femtosecond lamellar keratectomy, thus removing the infected tissue and increasing the drug delivery to the ulcer. Administration of polymyxin B/trimethoprim and tobramycin was successful in treating the ulcer with minimal residual scarring.

## Conclusions

*Nocardia* is a rare cause of keratitis. The SCUT study described *Nocardia* as the third causative agent of bacterial keratitis [47]. One study reported *N. arthritidis* and *N. asteroides* as the most important causative species of *Nocardia* keratitis, respectively [39]. *Nocardia* grow slowly in culture medium. Confocal microscopy and nucleic acid amplification are the newer diagnostic tools for *Nocardia*. Several antibiotics were used in the treatment of *Nocardia* keratitis, but fortified amikacin eyedrop remains the current best choice. Early diagnosis and treatment are essential to preserve good visual outcome; irreversible scarring may warrant additional surgical options. Adjuvant topical steroids should be avoided in the management of *Nocardia* keratitis.

## Data Availability

Not applicable.

## Abbreviations

PRK: Post Photorefractive Keratectomy
LASIK: LAser in SItu Keratomileusis
CED: Corneal Epithelial Defect
KPs: Keratic Precipitates
UV: Ultra Violet
GMS: Gomori Methenamin Staining
NGS: Next-Generation Sequencing
SCUT: Steroids for Corneal Ulcers Trial
BCVA: Best Corrected Visual Acuity
AMT: Amniotic Membrane Transplantation
PED: Persistent Epithelial Defect
